# A Substitute for Celluloid

**Published:** 1883-09

**Authors:** 


A Substitute for Celluloid.—A new material has been invented which, according to the Tradesman, it is thought will supersede celluloid. It possesses all the hardness and brilliancy of the latter, and has the advantage of being fire proof. It is made in this way: A solution is prepared of 200 parts of casein in 50 parts of ammonia and 400 of water, or 150 parts of albumen in 400 parts of water. To the solution the following are added: quick lime, 240 parts; acetate of alumina, 150 parts; alum, 50 parts; sulphate of lime, 1,200 parts; oil, 100 parts. The oil is to be mixed in last. When dark objects are to be made, from 75 to

100 parts of tannin are substituted for the acetate of alumina. When the mixture has been well kneaded together and made into a smooth paste, it is passed through rollers to form plates of the desired shape. These are dried and pressed into metalic moulds previously heated, or they may be reduced to a very fine powder, which is introduced into heated moulds and submitted to a strong pressure. The objects are afterward dipped into the following bath: Water, 100 parts; white glue, 6 parts; phosphoric acid, 10 parts. Finally they are dried, polished, and varnished with shellac.

